# Targeting the glycans of the HIV envelope as a therapeutic tool

**DOI:** 10.1186/1742-4690-8-S2-O38

**Published:** 2011-10-03

**Authors:** Jan Balzarini

**Affiliations:** 1 Rega Institute for Medical Research, K.U.Leuven, B-3000 Leuven, Belgium

## 

Carbohydrate-binding agents (CBA) represent a broad family of agents that recognize specific glycan conformations. Several members of this compound family including peptidicprokaryotic, plant, invertebrate and vertebrate lectins, but also the well-defined non-peptidicpradimicin and benanomicin antibiotics have shown to be endowed with anti-HIV activity. These compounds inhibit entry of virus particles in susceptible T-lymphocytes and macrophages, block syncytia formation between HIV-infected and non-infected T-lymphocytes, prevent capture of HIV by DC-SIGN-expressing cells and by the macrophage mannose receptor and prevent subsequent transmission of captured HIV to susceptible T-lymphocytes. No other antiviral class of compounds have been demonstrated to block these four important ways of HIV infection/transmission, which make the CBAs an interesting new family of potential anti-HIV drugs that may be particularly useful for both systemic and topical (i.e. microbicidal) applications. CBAs bind to the glycans of the HIV envelope gpl 20 and block viral entry, most likely by freezing the envelope conformation during viral entry. Escalating drug pressure in HIV-infected cell cultures result in the selection of mutant virus strains predominantly having one or several N-glycan deletions in their envelope gp120.ln this respect, many CBAs have a high genetic barrier since several N-glycan deletions seem to be required for significant phenotypic drug resistance. Interestingly, mutant virus strains containing an increasing amount of N-glycan deletions in gp120 tend to show a compromised ínfectivity potential. Moreover, we have found one specific, highly conserved N-glycan in HIV-1 gp120, whose deletion results in the production of noninfectious virus particles lacking gp41gp120 in their envelope. This N-glycan may be regarded as a hot-spot for a targeted therapeutic intervention. Also, it is expected that if mutant virus particles lacking N-glycans in gp120 are selected under CBA pressure *in vivo*, as shown to occur in drug-exposed HIV-and SIV-infected cell cultures, previously hidden conserved immunogenic epitopes become exposed, allowing the immune system to specifically trigger an efficient immune response against these viruses. An *in vivo* study using SIV-infected monkeys has been planned to explore this new therapeutic concept.

